# The Role of Autophagy in Osteoarthritis

**DOI:** 10.3389/fcell.2020.608388

**Published:** 2020-11-25

**Authors:** Ran Duan, Hui Xie, Zheng-Zhao Liu

**Affiliations:** ^1^Movement System Injury and Repair Research Center, Xiangya Hospital, Central South University, Changsha, China; ^2^Department of Sports Medicine, Xiangya Hospital, Central South University, Changsha, China; ^3^Department of Orthopedics, Xiangya Hospital, Central South University, Changsha, China; ^4^Hunan Key Laboratory of Organ Injury, Aging and Regenerative Medicine, Changsha, China; ^5^Hunan Key Laboratory of Bone Joint Degeneration and Injury, Changsha, China; ^6^National Clinical Research Center for Geriatric Disorders, Xiangya Hospital, Changsha, China

**Keywords:** apoptosis, autophagy, cartilage, chondrocyte, osteoarthritis

## Abstract

Chondrocytes are the only cell type in normal cartilage. The pathological changes of osteoarthritis (OA) mostly revolve around the apoptosis and dysfunction of chondrocytes. Autophagy, as an intracellular degradation system that maintains the steady state of energy metabolism in cells, has been shown to restore the function of damaged chondrocytes, alleviating the occurrence and progression of OA. In this review, we explored the relationship between autophagy and OA and the key molecules of autophagy pathway that regulate the progression of OA, providing new ideas for OA treatment by targeting autophagy.

## Introduction

OA is a chronic and high prevalent arthropathy and the most common cause of pain and disability worldwide characterized by progressive cartilage degradation, synovitis, and conversion of joint peripheral sclerotin including osteophyte formation and subchondral bone sclerosis (Harrell et al., 2019). A variety of risk factors contribute to the development of OA, such as age, obesity, hereditary factors, and preceding joint injuries (Vina and Kwoh, 2018). Senility is the greatest risk factor of OA (Jeon et al., 2017). The incidence of OA is rising due to the increasing obesity and a rapidly aging population. Over 50% OA patients are over 65 years old (Loeser, 2017), and even 80% of OA patients are over 75 years old (O'Neill et al., 2018). Articular cartilage is a kind of connective tissue composed of chondrocytes and extracellular matrix (ECM) that is synthesized and secreted by chondrocytes. The matrix that encapsulates chondrocytes plays a role of lubrication and mechanical support for cartilage joint. Although chondrocytes account for only 1% of the total cartilage volume, it plays an indelible role in maintaining the integrity of the matrix (Charlier et al., 2019; Rim et al., 2020). In fact, when certain factors act on the cartilage matrix and change its structure, chondrocytes can respond accordingly. However, the ability of articular chondrocytes to maintain the structure and integrity of normal cartilage matrix is limited and declines with aging (Martel-Pelletier et al., 2016). The existing treatment for OA is still relatively conservative, mostly limited to control the pain. However, in terms of reducing and controlling joint inflammation and promoting the recovery of damaged chondrocyte function, effective interventions that proven to alter the natural course of OA are still lacking (Cutolo et al., 2015; Hunter and Bierma-Zeinstra, 2019). Therefore, an in-depth investigation of the pathogenesis of OA is essential to explore new therapeutic targets.

Macroautophagy/Autophagy (hereafter referred to as autophagy), as a highly conserved degradation system, plays a vital role in the regulation of energy and nutrition, maintaining energy metabolism in the body (Nakamura and Yoshimori, 2017; Yu et al., 2018). Autophagy plays a protective role on cells under abnormal physiological conditions, including external pressure, hypoalimentation, hypoxia, endoplasmic reticulum stress (ERS), and so on. Malfunctioning cytoplasmic macromolecules, membranes and organelles are transported to lysosomes for degradation and reusing through autophagy pathway (Nakamura and Yoshimori, 2017). The occurrence of autophagy can be artificially divided into the following steps, including initiation, nucleation, elongation, maturation, and degradation. With the formation of these autophagosomes, degradation of chelate products provides amino acids, nucleotides, saccharides and aliphatic acids for maintaining the steady state of the entire tissue and even the body (Glick et al., 2010).

As mentioned above, since autophagy can degrade and remove long-lived or impaired organelles and proteins, the reduction of autophagy with aging is related to various aging diseases (Ren and Zhang, 2018). With the growth of age, the basic autophagic activity of cells decreases, following a down-regulated clearance efficiency. Subsequently, the aggregation of various macromolecular proteins increases, leading to the eventual cell degeneration and functional defect, or even apoptosis. In recent years, the inhibition of chondrocytes apoptosis by autophagy activation has attracted much attention (Shapiro et al., 2014). Recent studies have shown that the level of autophagy in OA cartilage is reduced (Feng et al., 2020), and autophagy can protect chondrocytes from degradation (Caramés et al., 2012). With the progress of OA, mammalian target of rapamycin (mTOR), the main negative regulator of autophagy, is up-regulated and mediates the inhibition of autophagy signal transduction in articular cartilage, and the protective effect of autophagy on cartilage decreases, eventually promoting cartilage degeneration (Vasheghani et al., 2015). Study by Zhang et al. demonstrated that the surgical destabilization of the medial meniscus (DMM) OA mouse model has higher level of autophagy, lower level of apoptosis when knocked out of mTOR (Zhang Y. et al., 2015). Activation of autophagy in chondrocytes by intra-articular injection of resveratrol, an autophagy inducer, can significantly delay articular cartilage degeneration of DMM OA mouse model (Qin et al., 2017). In recent years, studies on pharmacological suppression and gene deletion of mTOR to reduce the severity of OA have also repeatedly demonstrated the protective effect of autophagy on chondrocytes (Takayama et al., 2014; Ribeiro et al., 2016).

## Mechanistic Studies of Autophagy and OA

Association between aging and OA has been demonstrated in clinic and epidemiology. Risk factors related to aging, such as the limited ability of tissue and cell regeneration, increased expression of inflammatory mediators, oxidative stress, etc., cause damage to the cartilage matrix and cells, and promote the occurrence and development of OA (Rahmati et al., 2017). In recent years, autophagy, as a protective mechanism in cells, has attracted much attention because of its role in regulating the aging process. Autophagy will be activated when oxidative stress occurs, but excessive oxidative stress will exceed the tolerance of autophagy and impair its activity, eventually leading to cellular senescence and apoptosis (Roca-Agujetas et al., 2019). However, definitive studies on the relationship between autophagy and senescence are still lacking since some authors suggest a direct connection between autophagy and senescence and others indicative of an inverse relationship. Indeed, autophagy may promote or counteract senescence depending on the cellular context and stress stimuli (White and Lowe, 2009; Gewirtz, 2014). Study by Capasso et al. showed that in acute senescence, but low radiation, the autophagy flux is heavily impaired suggesting autophagy counteracts deteriorative processes, and its decline triggers senescence. This did not occur in replicative senescence. The authors hypothesized to reconcile these opposite events, that cells try to contend with stress by activating autophagy that eliminates damaged components. In that context, autophagy protects from senescence, and impairment of its function may promote senescence. On the other hand, if autophagy cannot counteract stress-induced damage, it may induce senescence (Capasso et al., 2015). In the state of excessive oxidative stress, chondrocytes can enhance autophagic activity and inhibit aging process by activating AMP-activated protein kinase (AMPK) or inhibiting mTOR (Han et al., 2016; Tai et al., 2017). Another important sign of aging is mitochondrial dysfunction associated with an increase of superoxide accumulation. It is noteworthy that mitochondrial phagocytosis can reduce phenotypes of cell senescence (Tai et al., 2017).

Inflammatory cytokines, mechanical stress and senescence can cause elevated levels of reactive oxygen species (ROS) in chondrocytes. Known ROS include superoxide, hydrogen peroxide, peroxyl radicals, the reactive nitrogen species (including nitric oxide and peroxynitrite derived from the nitric oxide) (Su et al., 2019). Kongara and Karantza have shown that autophagy is responsible for eliminating intracellular sources of ROS, including mitochondria and peroxisomes (Kongara and Karantza, 2012), thus, autophagy defect leads to accumulation of ROS (Scherz-Shouval and Elazar, 2007). Under pathological conditions, the phenomenon of cartilage degradation can be attributed to excessive ROS acting as second messengers, which subsequently mediate the inhibition of matrix synthesis, affect cell migration and the biological activity of growth factors, which in turn regulate the degradation of matrix components, activation of matrix metalloproteinases (MMP) and cell death. Excess ROS can cause inhibition of the mitochondrial respiratory chain, decrease of ATP, mitochondrial DNA (mtDNA) mutations, and disorder of redox regulated cell signaling pathways such as protein kinase B (PKB/AKT) and mitogen-activated protein kinase (MAPK) signaling pathways. ROS-induced mitochondrial damage and activation of endoplasmic reticulum (ER) stress play a key role in OA, and may eventually trigger the cascade of chondrocytes apoptosis (Collins et al., 2018; Bolduc et al., 2019). Previous studies have shown that both autophagy defect and mitochondrial dysfunction were found in age-related and surgically induced OA mouse models, and mitochondrial function and autophagy in chondrocytes are directly mediated by the AKT-mTOR signaling pathway (Barranco, 2015). Inhibition of autophagy related 5 (*Atg5*) in chondrocytes increases the production of ROS, and induces mitochondrial dysfunction (Lopez de Figueroa et al., 2015). These results indicated that autophagy participates in the occurrence and development of OA by mediating apoptosis and ROS production (Charlier et al., 2016). When autophagy is activated, the damaged mitochondria can be removed and intracellular ROS reduces, protecting chondrocytes from the negative effects of OA. Therefore, we have every reason to believe that autophagy plays an irreplaceable role in protecting chondrocytes from oxidative stress (Ansari et al., 2018).

## Key Molecules Involved in Autophagy and OA

**Figure 1:**
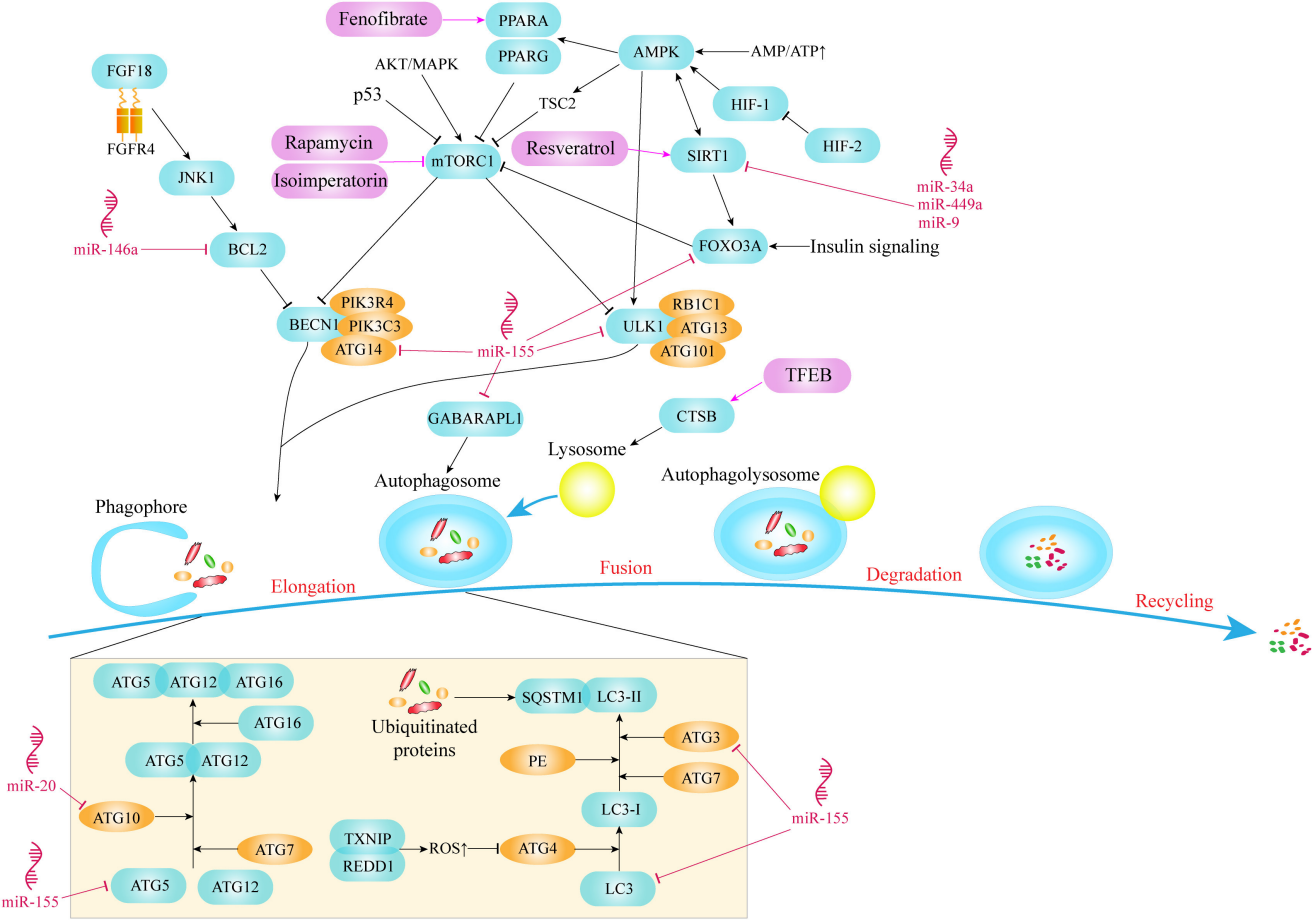
Key molecules and pathways of autophagy in OA.

mTOR kinase is a key molecule in the process of autophagy. It combines with various proteins to form two different multiprotein complexes known as mTOR complex 1 (mTORC1) and 2 (mTORC2). mTORC1, as a complex containing mTOR, plays a vital role in the regulation of autophagy (Laplante and Sabatini, 2012). Studies have shown that mTORC1 mainly acts as a negative regulator in autophagy and can be regulated by a variety of signaling molecules to affect autophagic activity (Choi et al., 2013). Thus, mTOR activation pathways, such as AKT and MAPK signaling pathways, inhibit autophagy, while mTOR negative regulation pathways, such as AMPK and p53 signaling pathways, promote autophagy. AMPK is an energy-sensing kinase, a highly conserved protein molecule during evolution. When metabolic stress or ATP consumption occurs, AMPK can be activated to promote catabolism. Once the AMPK pathway activated, AMPK phosphorylates tuberous sclerosis 2 (TSC2), which then inhibits mTOR and eventually promotes autophagy. In addition, AMPK can also directly regulate autophagy by acting on downstream signaling molecules of mTOR (Alers et al., 2012). In summary, both mTOR and AMPK are integral parts of autophagy initiation (Figure 1).

The chondrocytes in the growth plate survive in a microenvironment with almost no blood vessels and hypoxia, and have evolved a different way of energy generation, generating metabolic energy through anaerobic glycolysis. The hypoxia-inducible factors, HIF-1 and HIF-2, mediate the acclimatization of chondrocytes to this avascular environment (Zhang F.-J. et al., 2015). HIF-1α is highly expressed in hypertrophic chondrocytes (Taheem et al., 2018). Activation of HIF-1 enhances the autophagic activity of chondrocytes in hypoxia condition. Reduction of energy charge activates HIF-1, which leads to the phosphorylation and activation of AMPK. AMPK enhances autophagy flux by inhibiting mTOR and promoting other downstream signaling molecules (Bohensky et al., 2010). In contrast with HIF-1, HIF-2 is a potent negative regulator of autophagy in maturing chondrocytes which is elevated in OA cartilage and drives catabolic metalloproteinases, such as MMP-13 which is a major enzyme that mediates the degradation of type II collagen in cartilage (van den Berg, 2011; Wang et al., 2013) (Figure 1).

As a core protein with serine/threonine kinase activity in autophagy signaling pathway, unc-51 like autophagy activating kinase 1/2 (ULK1/2) can form the ULK complex with RB1 inducible coiled-coil 1 (RB1CC1/FIP200), autophagy related 13 (ATG13) and autophagy related 101 (ATG101) and mediate the activation of autophagy signaling pathway (Mizushima, 2010). ULK1 in a phosphorylation status has always been identified as a pivotal sign of autophagy initiation. Phosphorylation of ULK1 was mediated by AMPK and mTOR signaling pathway (Figure 1). A large number of studies have shown that with the change in the nutritional status of tissues or cells, the phosphorylation status of the ULK1/2-ATG13-RB1CC1 complex will also undergo corresponding changes. Specifically, in the context of glucose starvation, AMPK directly phosphorylates ULK1 at Ser 317 and Ser 777 to promote autophagy (Shang et al., 2011). However, in the case of sufficient nutrition, the high activity state of mTOR phosphorylates ULK1 at Ser 757, destroys the mutual effect between ULK1 and AMPK, and finally prevents the activation of ULK1 (Kim et al., 2011).

One of the initial steps in assembling pre-phagocytic structures into autophagosomes is to recruit and activate the class III phosphatidylinositol 3-kinase complex which is composed of Beclin1 (BECN1), phosphoinositide-3-kinase regulatory subunit 4 (PIK3R4/VPS15), phosphatidylinositol 3-kinase catalytic subunit type 3 (PIK3C3/VPS34), and autophagy related 14 (ATG14/ATG14L) proteins (Menon and Dhamija, 2018) (Figure 1). The pivotal role of ATG14 in autophagy is the recruitment of autophagy specific PI3 kinase complex in endoplasmic reticulum (Matsunaga et al., 2010). Meanwhile, PIK3C3, as a kind of catalytic phosphatidylinositol 3 kinase, catalyzes the phosphorylation of phosphatidylinositol to form phosphatidylinositol 3-phosphate (PI3P). PI3P on the autophagosome membrane has the ability to recruit the membrane-bound protein ATG18 and bind to the bilayer membrane, which is crucial for the elongation and formation of autophagosomes (Noda et al., 2010).

In addition to the above autophagy pathway signaling molecules, two protein conjugation systems are also involved in the formation and amplification of autophagosomes, among which ATG proteins microtubule associated protein light chain 3 (LC3) and ATG12 are important components of the related protein coupling system. LC3 is usually used as a marker to evaluate the degree of autophagy. During the process of autophagy, ATG4 can remove the C-terminus of LC3 to generate LC3-I in the cytoplasm, and LC3-I is connected with phosphatidylethanolamine (PE) in the way of ubiquitin like reaction, which requires the participation of E1-like enzyme ATG7 and E2-like enzyme ATG3. LC3-II is transformed from LC3-I through the lipidation of the ubiquitin-like reaction described above and binds to autophagic vesicles (Li et al., 2020). Therefore, the amount of LC3-II is related to the degree of autophagosome formation, and the ratio of LC3-II/LC3-I is usually used to assess the level of autophagy (Martin-Rincon et al., 2018). In selective autophagy, cytoplasmic components that are selectively degraded are labeled with ubiquitin in order to be recognized by means of autophagy receptors before being sequestered into an autophagosome (Lamark et al., 2017). As a multifunctional adaptor protein, sequestosome 1 (SQSTM1/p62) is able to combine with mono- or poly-ubiquitinated proteins. Through its LC3-interacting region (LIR) motif, SQSTM1 directly binds to LC3 and becomes a selective autophagy receptor which aggregates ubiquitinated proteins and brings them into the emerging autophagosomes (Shaid et al., 2013). SQSTM1 also acts as cargo receptor and is degraded by autophagy together with ubiquitinated substrate proteins (Figure 1).

Conjugation of ATG5 and ATG12 is an essential link in autophagy (Figure 1). The genetic ablation of *Atg5* leads to the development of OA with age and the apoptosis of chondrocytes mediated by caspases (Vuppalapati et al., 2015; Bouderlique et al., 2016). As a ubiquitin activating enzyme, ATG7 plays an important role in autophagy formation and LC3 activation. Knocking out *Atg7* in chondrocytes leads to growth retardation of chondrocytes, which is also related to the reduction of chondrocyte proliferation and the upregulation of apoptosis (Kang et al., 2017; Horigome et al., 2020).

The defects of autophagy in OA cartilage include the decrease of the number and size of autophagosomes, which is related to the decreased expression of ULK1, BECN1, LC3 and the overexpression of mTOR (D'Adamo et al., 2017b). At the same time, SQSTM1 increased in OA cartilage, indicating that autophagy was inhibited (Zheng et al., 2019).

## Key Molecules Regulated the Autophagy Pathway in OA

### MicroRNAs Regulated the Autophagy Pathway in OA

MicroRNAs (miRNAs) are a type of non-coding small RNAs that promote specific mRNA degradation and/or translation inhibition, and play a pivotal role in biological processes as new gene expression regulators, whose regulatory mechanism is thought to be through sequentially specific interactions with the 3' untranslated regions (UTRs) of specific mRNA targets (Bartel, 2004). miRNAs play a vital role in cartilage homeostasis as well (Miyaki and Asahara, 2012). There are a lot of evidences revealing that miRNAs targeting autophagy pathway act as key regulators in the occurrence and development of OA (Al-Modawi et al., 2019; Yu and Zhao, 2019).

miR-155 is one of the most upregulated miRNAs in human OA cartilage. Study by Adamo et al., revealed that miR-155 plays an inhibitory role in autophagy of chondrocytes and explored its targets in autophagy pathway, including ULK1 participated in the startup phase of autophagy, ATG14 responsible for recruiting autophagy-specific PI3 kinase complex, ATG5, ATG3, LC3, and GABA type A receptor associated protein like 1 (GABARAPL1/ATG8L) involved in the formation of autophagosomes, and forkhead box O3 (FOXO3) which is the key transcription factor of autophagy-related genes (D'Adamo et al., 2016) (Figure 1).

In addition, other studies explored the regulatory effect of miRNAs on sirtuin1 (SIRT1) which is another crucial signaling molecule of autophagy pathway. MiR-34a, miR-449a and miR-9 have been demonstrated to significantly reduce the expression of SIRT1 in chondrocytes (Figure 1). Inhibition of miR-34a, or miR-449a, or miR-9 can alleviate cartilage damage by upregulating the expression of SIRT1, indicating an improved prognosis of OA (Park et al., 2016; Yan et al., 2016; D'Adamo et al., 2017a).

miR-140-5p and miR-149 could target fucosyltransferase1 (FUT1). An *in vitro* experiment proved that overexpression of miR-140-5p or miR-149 can down-regulate the intracellular level of FUT1, promoting survival of chondrocytes by activating autophagy and inhibiting the apoptosis (Wang et al., 2018). Moreover, there are many other miRNAs that affect chondrocyte function by targeting autophagy pathway, such as miR-20 affects chondrocyte function by targeting ATG10 (He and Cheng, 2018), miR-146a enhances autophagic activity of chondrocyte by decreasing the expression of BCL2 which is an autophagy inhibitor (Chen et al., 2017) (Figure 1).

Although the autophagy control mechanism has been extensively studied, the changes of miRNA in chondrocytes are still in sore need of further research to explore the autophagy control network. So far, interfering the gene expression with miRNA in chondrocytes has been proved a promising strategy for the treatment of OA.

### Other Key Molecules Regulated the Autophagy Pathway in OA

Based on the results of cell experiments, animal models of OA induced by various drugs or surgeries have been developed to study the causes and pathogenesis and to test some new therapeutic interventions. To explore the role of autophagy in cartilage of OA, mice specifically knocked out autophagy-related genes in cartilage were generated.

Cinque et al. discovered that the underlying mechanism of mild growth retardation after *Atg7* gene ablation involves impaired type II collagen formation and secretion in cartilage. And they demonstrated that induction of chondral autophagy after birth is mediated by the fibroblast growth factor 18 (FGF18) through fibroblast growth factor 4 (FGFR4) and the autophagy initiation complex VPS34-BECN1 (Cinque et al., 2015) (Figure 1). This suggests that FGF18 may be a key molecule that regulates autophagy signaling pathways during OA generation and development.

Gene ablation of mTOR, an effective negative regulator of autophagy in cartilage can enhance autophagic activity, significantly reduce the degradation, apoptosis and synovium fibrosis of articular cartilage in DMM induced OA model, and effectively maintain joint homeostasis (Zhang Y. et al., 2015). Knocking out peroxisome proliferator activated receptor gamma (*Pparg*) in mouse cartilage accelerates the development of OA. As a ligand-activated transcription factor, the lack of PPARG in articular cartilage leads to an up-regulation of mTOR in articular cartilage, which subsequently suppresses the level of autophagy and is associated with increased apoptosis of chondrocytes (Vasheghani et al., 2015) (Figure 1).

DNA damage inducible transcript 4 (DDIT4/REDD1) can be induced by hypoxia and other stresses. The specific knockout of *Ddit4* in a mouse model showed dramatically reduced LC3 and ATG5 expression levels, significant decrease of mtDNA, an increase apoptosis of chondrocytes in articular cartilage (Alvarez-Garcia et al., 2016, 2017). REDD1 has been shown to combine with thioredoxin interacting protein (TXNIP, a pro-oxidant protein) to form a protein complex in chondrocytes to induce the production of ROS, which is crucial for the induction of autophagy (Qiao et al., 2015). When the expression level of REDD1 in chondrocytes is reduced, ROS in the cytoplasm is down-regulated, which subsequently leads to the over-activation of cysteine endopeptidase ATG4, leading to delipidation of LC3 and defective autophagosome assembly (Figure 1). Therefore, we believe that REDD1 is the main molecule mediating articular cartilage homeostasis through autophagy pathway.

Deletion of *Foxo* transcription factors also produced OA-like changes (Matsuzaki et al., 2018). FOXO transcription factors could enhance the strength of chondrocytes to resist oxidative stress. Down-regulation of FOXO reduces the levels of autophagy-related proteins, such as LC3-II and BECN1 (Akasaki et al., 2014). Study also shows that the positive effect of FOXO3A in protecting chondrocytes by activating AMPK and autophagy pathway (Zhao et al., 2014) (Figure 1). FOXO transcription factors are also demonstrated as downstream effectors of insulin signaling under low nutrient situations, activating the transcription of glutamine synthetase and inhibiting mTOR to enhance autophagic activity (Webb and Brunet, 2014). *Sirt1* haploid deficiency mouse model showed a slow-growing phenotype and was accompanied by spontaneous OA at 9 months, which may be related to the change in the number of chondrocytes (Gabay et al., 2012). Mice knocking out *Sirt1* in chondrocytes develop OA in a time-dependent manner (Matsuzaki et al., 2014). Previous study also revealed that SIRT1 regulates autophagy and plays a protective role on chondrocytes through its effect on FOXO, as downstream of SIRT1 which is a conserved family of NAD^+^-dependent deacetylases, are the FOXO family members (Almeida and Porter, 2019) (Figure 1).

It has been shown that mouse models of OA are essential to improve our understanding of the potential molecular mechanisms of OA.

## Status of OA Therapeutics Targeting Autophagy Signaling Pathway

To delay the progress of OA clinically, various treatments have been developed, including medication, non-drugs therapy and physical therapy. Medical treatment includes some conventional drugs such as non-steroidal anti-inflammatory drugs, but these drugs have serious side effects, including damage to the gastrointestinal tract and increased susceptibility to cardiovascular and kidney diseases. Non-pharmacologic treatments consist of weight loss, biomechanical intervention, electromagnetic stimulation, and shock wave therapy (Cooper et al., 2019). Surgical treatment is a routine treatment for advanced OA, usually total joint replacement (Nelson, 2018; Kloppenburg and Berenbaum, 2020).

The current treatment methods are limited to symptomatic treatment to delay the course of OA. Various new drugs developed have no obvious benefit or have serious side effects and do not applicable in clinic. As an important role in the pathogenesis of OA, autophagy has been attracted intensive attention to be a target for new OA treatment. The development of safe and effective drugs that can enhance autophagic activity or restore autophagy flux is a promising strategy for the treatment of OA.

mTOR, as a key signaling molecule in autophagy pathway, has become an important target of drugs targeting autophagy pathway. Rapamycin has been proved to reduce the expression of mTOR and delay the degradation of articular cartilage. Local application of rapamycin by intra-articular injection may be a potential therapy for OA (Takayama et al., 2014). Other drugs such as isoimperatorin and glucosamine can also activate autophagy and improve cell homeostasis by inhibiting mTOR pathway (Ouyang et al., 2017) (Figure 1). In addition, isoimperatorin improve the pathological changes induced by OA by reducing the expression of matrix metallopeptidase 13 (MMP13), RUNX family transcription factor 2 (RUNX2), collagen type X alpha 1 chain (COL10A1) and vascular endothelial growth factor A (VEGFA). However, Glucosamine shows a dual role in dose-dependent and time-dependent manner in the regulation of chondrocyte survival and apoptosis. This is because when chondrocytes are exposed to glucosamine for a short period of time, autophagic responses will be activated (called a short-term response), and long-term exposure will cause the suppression of the level of autophagy, especially pexophagy and peroxidative damage (called a long-term response) (Kang et al., 2015). Resveratrol can activate SIRT1 in order to inhibit OA disease progression (Deng et al., 2019) (Figure 1).

Drugs regulating other targets, such as Tougu Xiaotong Capsule, facilitate autophagy by balancing ATG12/LC3 coupling system (Li et al., 2014). Several autophagosome formation indexes including ATG3, ATG7, ATG12-5 and BECN1 in trehalose treated chondrocytes and the proportion of LC3-II:LC3-I increased as the dose of the drug increases, and the level of SQSTM1 decreased, indicating that trehalose can enhance autophagy flux (Tang et al., 2017). Fenofibrate (FN) as a PPARA agonist is commonly used to treat abnormal blood lipid levels in humans. Studies have found that FN can reduce the number of senescent cells, increase autophagy level and prevent cartilage degradation. The abnormal appearance of cartilage after *Ppara* knockdown also verified the role of FN (Nogueira-Recalde et al., 2019) (Figure 1). Transcription factor EB (TFEB) is known as a member of melanocyte inducing transcription factor (MITF)/transcription factor E (TFE) family. TFEB has indeed been proved to be the main regulator of autophagy level. It can induce the occurrence of lysosomes, promote the formation of autophagosomes and mediate their fusion (Settembre et al., 2011). The mechanism may be that on the one hand, increase the formation of LC3-II to promote the process of autophagy, on the other hand, up-regulate the expression of cathepsin B (CTSB) and increase the acidity of lysosomes to restore the function of lysosomes, ultimately rescuing the destruction of autophagy flux (Zheng et al., 2018) (Figure 1).

## Conclusion

As an age-related disease, the incidence of OA increases with aging. In recent years, the basic researches of OA from various countries have centered around the age-related changes of chondrocytes. These changes include not only damaged proteins and abnormal accumulation of lipids, but also changes in ROS levels caused by impaired mitochondrial function and changes in autophagy and energy metabolism caused by oxidative stress. These subcellular disorders, on the one hand, destroy the relevant signaling pathways and normal function of cells, on the other hand they also promote catabolic activity and eventually lead to apoptosis of chondrocytes.

In the pathogenesis of OA, apoptosis and autophagy are considered to be two important links. At present, it is generally believed that autophagy as an adaptive response can reduce cell death in the early stage of OA, but with the development of OA, excessive autophagy may also cause cell death. Even so, we believe that the activation of autophagy has a positive significance for the survival of chondrocytes in the early development of OA. Although the role of autophagy disorder in the pathogenesis of OA has not been fully elucidated, autophagy as a therapeutic target for OA still shows broad clinical prospects. The targeted application of small molecule drugs to regulate the level of chondrocyte autophagy is expected to provide more options for the clinical treatment of OA.

## Author Contributions

RD wrote the manuscript. HX and Z-ZL guided the planning, writing of the review manuscript, and critically amended the manuscript. All authors read and approved the final manuscript.

## Conflict of Interest

The authors declare that the research was conducted in the absence of any commercial or financial relationships that could be construed as a potential conflict of interest.
